# Compatible Solute Engineering in Plants for Abiotic Stress Tolerance - Role of Glycine Betaine

**DOI:** 10.2174/1389202911314030001

**Published:** 2013-05

**Authors:** Shabir Hussain Wani, Naorem Brajendra Singh, Athokpam Haribhushan, Javed Iqbal Mir

**Affiliations:** 1 Farm Science Centre-(KVK) Hengbung, Senapati, Manipur, India 795129; 2 Department of Plant Breeding and Genetics, COA, Central Agricultural University, Imphal, Manipur, India 795004; 3 Biotechnology Laboratory, Central Institute of Temperate Horticulture, Rangreth, Srinagar, Jammu and Kashmir 190007, India

**Keywords:** Glyicine betaine, Abiotic stress, Osmoprotectants, Salinity, Compatible solute, Genetic engineering, Choline.

## Abstract

Abiotic stresses collectively are responsible for crop losses worldwide. Among these, drought and salinity are the most destructive. Different strategies have been proposed for management of these stresses. Being a complex trait, conventional breeding approaches have resulted in less success. Biotechnology has emerged as an additional and novel tool for deciphering the mechanism behind these stresses. The role of compatible solutes in abiotic stress tolerance has been studied extensively. Osmotic adjustment, at the physiological level, is an adaptive mechanism involved in drought or salinity tolerance, which permits the maintenance of turgor under conditions of water deficit, as it can counteract the effects of a rapid decline in leaf water potential. Increasing evidence from a series of *in vivo* and *in vitro* studies of the physiology, biochemistry, genetics, and molecular biology of plants suggest strongly that Glycine Betaine (GB) performs an important function in plants subjected to environmental stresses. It plays an adaptive role in mediating osmotic adjustment and protecting the sub-cellular structures in stressed plants, protection of the transcriptional and translational machineries and intervention as a molecular chaperone in the refolding of enzymes. Many important crops like rice do not accumulate glycinebetaine under stress conditions. Both the exogenous application of GB and the genetically engineered biosynthesis of GB in such crops is a promising strategy to increase stress tolerance. In this review we will discuss the importance of GB for abiotic stress tolerance in plants. Further, strategies like exogenic application and transgenic development of plants accumulating GB will be also be discussed. Work done on exogenic application and genetically engineered biosynthesis of GB will be listed and its advantages and limitations will be described.

## INTRODUCTION

Environmental stress severely restricts the distribution and productivity of plants. In particular, salinity and drought are two major constraints that limit agricultural production world‐wide [1-5]. Plants have evolved various protective mechanisms that allow them to acclimate to unfavourable environments for continued survival and growth. One such mechanism that is ubiquitous in plants is the accumulation of certain organic metabolites of low molecular weight that are known collectively as compatible solutes [6]. Osmoprotectants or compatible solutes are small molecules that act as osmolytes and help organisms survive extreme osmotic stress [7]. Examples include betaines, amino acids, and the sugar trehalose. These molecules accumulate in cells and balance the osmotic difference between the cell's surroundings and the cytosol. Osmoprotectants are small neutral molecules that are non- toxic to the cell at molar concentration and that stabilize proteins and cell membranes against the denaturing effect of stress conditions on cellular functions [8]. Many major crops lack the ability to synthesize the special osmoprotectants that are naturally accumulated by stress-tolerant organisms. It has been hypothesized, therefore, that engineering the introduction of osmoprotectant synthesis pathways is a potential strategy for improving the stress tolerance of crop plants [9].

The metabolites which act as compatible solutes are different among various species of plants and include polyhydroxylated sugar alcohols, amino acids and their derivatives, tertiary sulphonium compounds and quaternary ammonium compounds [10]. There is general agreement that the major role of these metabolites is to serve as organic osmolytes with compatible properties at high concentrations; such osmolytes increase the ability of cells to retain water without disturbing normal cellular functions [11]. Metabolic acclimation via the accumulation of compatible solutes is often regarded as a basic strategy for the protection and survival of plants under abiotic stress [10, 12-15].

Glycinebetaine (*N*,*N*,*N*‐trimethylglycine) is widely available in various varieties of plants, animals and micro-organisms [16-19]. At physiological pH, it is a dipolar but electrically neutral molecular. In the case of osmoregulation, the compatible solute glycine betaine (GB), a small organic metabolite soluble in water and non-toxic at high concentrations, is a compound that can potentially play a crucial role in effective protection against salt, drought, and extreme temperature stress [19-21]. Accumulation and biosynthesis of Glycine betaine has been thoroughly investigated among the halophyte species [16, 22, 23]. The major role of GB in plants exposed to saline soil is probably protecting plant cells from salt stress by osmotic adjustment [24], protein stabilization (RuBisCo) [25], photosynthetic apparatus protection [26], and reduction of oxygen radical scavengers [27].

With respect to protection against osmotic stress, GB is regarded as being a particularly effective compatible solute [28]. Numerous experiments *in vitro* have indicated that betaine acts as an osmoprotectant by stabilizing both the quaternary structure of proteins and the highly ordered structure of membranes against the adverse effects of high salinity and extreme temperatures [29]. It accumulates to osmotically significant levels in many salt-tolerant plants [16] and halotolerant cyanobacteria [19].

The accumulation of betaine in response to salt, drought and cold has been widely recognized in higher plants that are natural accumulators of this compound [29]. Plants in many taxonomically distant species normally contain low levels of GB (these plants are known as natural accumulators of GB), but they accumulate larger amounts of GB when subjected to abiotic stress [30]. In many other species, no GB is detectable under normal or stressful conditions. 

Some economically important crops, including rice (*Oryza sativa*), potato (*Solanum tuberosum*), and tomato (*Solanum lycopersicum*), are unable to accumulate GB; therefore, these species are potential targets for engineering of betaine biosynthesis [31].

With increasing knowledge of genomics and proteomics coupled with gene engineering technologies, several plant species have been engineered with genes of the GB biosynthetic pathway that confer tolerance to several abiotic stresses [32]. There is now strong evidence that GB plays an important role in tolerance to abiotic stress.

## BIOSYNTHESIS OF GLYCINEBETAINE

**Fig. (1):**
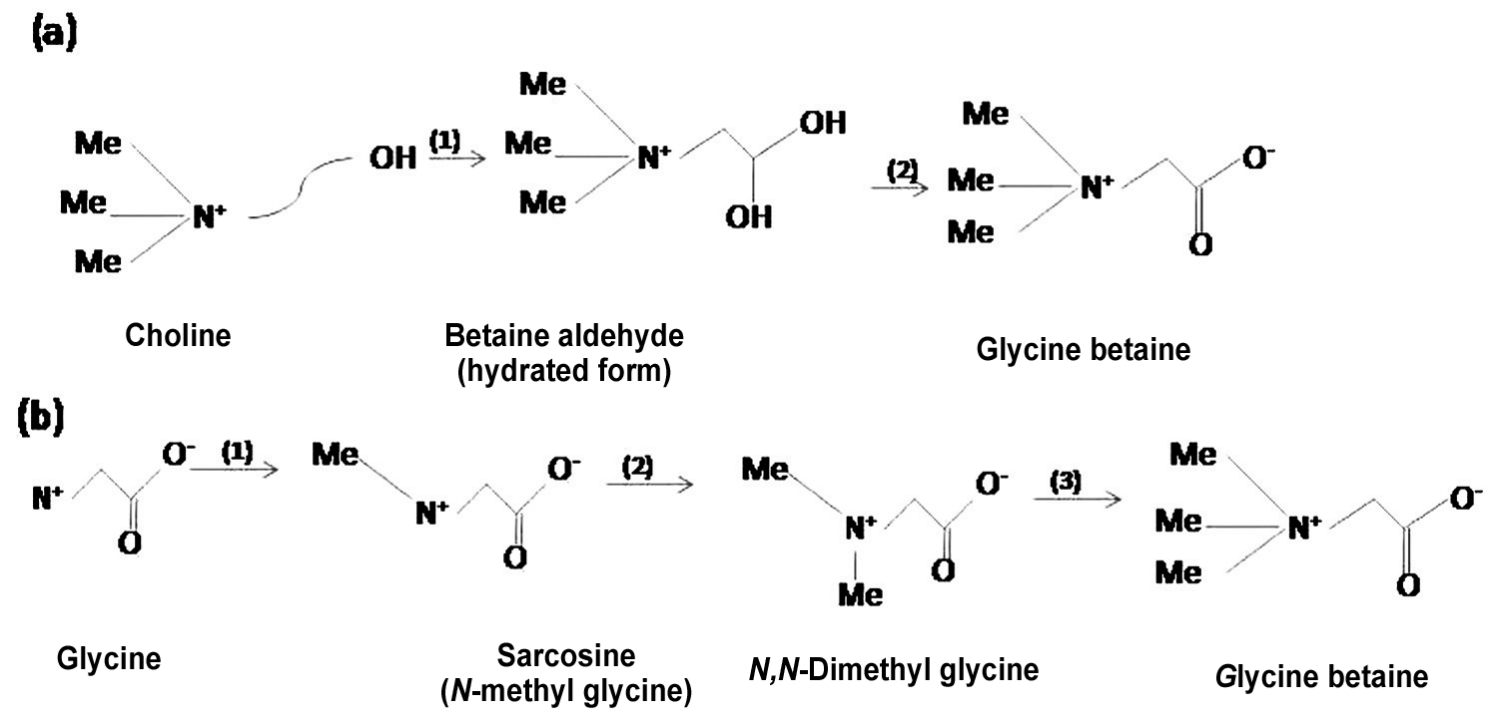
The two main pathways for the synthesis of GB Source: -Sakamoto and Murata [33].

In known biological systems, GB is synthesized via two distinct pathways from two distinct substrates: choline and glycine, respectively [33] (Fig. **1**). The conversion of choline to GB has been studied in a number of organisms and the pathway involves one or two enzymes, depending on the mode of oxidation of choline (Fig. **1a**). The two-enzyme pathway is widespread, occurring naturally in various plants, animals and micro-organisms. In this pathway, GB is formed as the result of the two-step oxidation of choline via the toxic intermediate betaine aldehyde. In higher plants, the reactions are catalysed by choline monooxygenase (CMO) and NAD^+^-dependent betaine aldehyde dehydrogenase (BADH), both of which are localized in the stroma of chloroplasts. The biosynthesis of GB is stress-inducible and the concentration of GB *in vivo* varies among plant species, ranging from 40 to 400 μ mol (g DW) ^-1^ in so called natural accumulator under stress conditions [16].

Certain bacteria, for example *Escherichia coli*, are capable of producing GB from choline via the actions of two dehydrogenases: a membrane-bound CDH and BADH [34]. Animal cells can synthesize GB via the same CDH/BADH pathway [35]. In contrast the single-enzyme pathway has only been recognized in soil bacteria in the genus *Arthrobacter* to date [36]. The entire reaction is catalysed by COD, an H_2_O_2_-generating oxidase. The pathway from glycine to GB was discovered only recently and, to date, it has been recognized in only two extremely halophilic micro-organisms, *Ectothiorhodospira* *halochloris* and *Actinopolyspora halophilia* (Fig. **1b**), [37]. In these micro-organisms, GB is generated from glycine via three successive *N* -methylations, which are catalysed by two *S* -adenosylmethionine-depen dent methyl transferases, glycinesarcosine methyltransfe rasec(GSMT) and sarcosine dimethylglycine methyltransferase (SDMT) [17, 18].

The genes for all the enzymes involved in the various pathways have been cloned and they are available for the metabolic engineering of plants that do not accumulate GB naturally, thus endowing them with the potential ability to synthesize this important compound.

## ACCUMULATION OF GB IN PLANTS

Glycine betaine (GB) is a quaternary ammonium compound that occurs in a wide variety of plants, but its distribution among plants is sporadic [38]. Arabidopsis and many crop species do not accumulate GB. Some higher plants are not capable to accumulating GB [39]. For example, tomato plants (*Lycopersicon* *esculentum *Mill.) do not naturally accumulate GB [40].

Plants in many taxonomically distant species normally contain low levels of GB (these plants are known as natural accumulators of GB), but they accumulate larger amounts of GB when subjected to abiotic stress [30]. In many other species, no GB is detectable under normal or stressful conditions. Glycinebetaine (GB) accumulates in a variety of organisms under abiotic stresses and has been studied in great details [10, 17]. In plants that produce GB naturally, abiotic stress, such as cold, drought, and salt stress, enhances GB accumulation [16, 41].

The biological functions of GB have been studied extensively in higher plants, such as spinach, sugar beet, barley and maize [16, 19]. Many lines of GB-accumulating transgenic plants exhibit greatly improved tolerance to various types of abiotic stress and their properties suggest promising strategies for the development of stress-tolerant crop plants.

Several reviews of the relationship between GB and tolerance to abiotic stress have appeared recently [13, 17-19] but progress in the field has been rapid and a fresh appraisal is appropriate.

## EXOGENEOUS APPLICATION OF GB IN PLANTS ENHANCING ABIOTIC STRESS

There is now strong evidence that GB plays an important role in tolerance to abiotic stress. Exogenous application of GB to non-accumulator plants may be a possible alternative, approach for tolerance against multiple abiotic stresses [39]. Among the different betaines naturally occuring in plants, GB accumulates in many families like, the Asteraceae, Chenopodiaceae, Poaceae and Solanaceae [40] in response to abiotic stresses [42]. All the higher plants do not have the capacity to accumulate GB [39]. Evidence reveals that soybean is normally a low-accumulator of GB [43], with an average content less than 5 μmol/g dry weights [44]. Foliar application of GB could increase its content in soybean plant up to 60 μmol/g dry weights, leading to an improvement in photosynthesis activity, nitrogen fixation, leaf area development, and seed yield of both well irrigated and drought-stressed soybean plants [45, 46]. The accumulated GB may maintain cellular osmotic balance [31] and stabilizes quaternary structures of complex proteins [47]. Reports by Larkum and Wyn Jones [48] and Smirnof and Stewart [49] indicated that GB had a positive influence on key factors contributing to economic yield of plants under stress.

Salt stress up regulates the enzymes involved in proline and betaine biosyntheses in several plant species [50, 51], and elevated levels of proline and betaine accumulated in plant cells correlate with enhanced stress tolerance [52-54]. For example, foliar application of GB improved salt and drought tolerance in tomato [55, 56]. Uptake of foliar applied GB has been shown to be active [57]. Exogenous GB was absorbed by the leaves and remained stable which improves the growth, survival, and tolerance of a wide variety of accumulator/non-accumulator plants under various stress conditions [58-60]. Ashraf and Foolad, [21] suggested that effectiveness of foliar applied GB depends on a number of factors including plant species, plant developmental stage at which GB applied, concentration of GB, and number of applications. It is suggested that GB is not a compatible organic osmoticum for all plants or cause of phytotoxicity, when applied either at higher concentration or by increasing the number of applications. The broadleaf species such as bean, tomato, and grape are more sensitive to high concentrations of GB than grass species/cereals. Therefore, it is important to determine optimal concentration of GB, number of applications, and time of application for each crop species [61].

There are many reports demonstrating positive effects of exogenous application of GB on plant growth and final crop yield under drought stress; examples include those in tobacco, wheat, barley, sorghum and soybean [21]. Rezaei *et al.* [62] indicated the role of exogenous glycine betaine in reduction of harmful effects of drought stress and its useful effects on yield and yield components. Foliar application of tomato plants in field condition with 3.36 Kg ha^-1^ GB during mid flowering period increased fruit yield 36 and 39%, as compared to control in salt and heat stress, respectively [55]. GB applied at the vegetative growth stage was more effective in ameliorating the adverse effects of drought stress on tomato cv. PS, due to GB-induced improvement in plant water status. The adverse effects of drought stress on tomato can be alleviated by the exogenous application of GB at different growth stages by modulating water relations [63].

Studies with cereals showed contradictory results. Thai jasmine rice cultured under salt-stress condition supplemented with GB and Cho in the culture media directly enhanced the accumulation of GB, in the context of whole plant physiology, pigment stabilization for water oxidation of photochemistry PSII as well as CO_2_- assimilation. The water oxidation and CO_2_ assimilation of GB-accumulated seedlings cultured under salt stress condition positively related to NPR as well as growth promotion. The exogenous application of GB and Cho in the culture media would provide an alternative way for improving the defense mechanisms of plants to salt stress [64]. Rice has been reported as being a GB non-accumulator, while GB has been detected in small amounts in rice cultivars exposed to salt stress, including KDML105 [65], Homjan [66], and Annapurna and Dongjin [67]

Agboma *et al.* [43] concluded that the application of GB, in field conditions, improved drought tolerance and increased the yield of maize and sorghum, but not for wheat. These researchers found that Exo-GB delayed the canopy senescence of barley, oat and wheat, but these differences were not associated with differences in grain yields [44].

## GENETIC ENGINEERING OF PLANTS FOR ACCUMULATION OF GB

Various genes have been used to generate transgenic plants that accumulate GB and exhibit enhanced tolerance to abiotic stress. Among such transgenic plants, *codA *transgenic plants have been investigated most thoroughly in terms of the physiological and morphological aspects of stress tolerance at all stages of the life cycle of plants, from imbibition of seeds, through the growth of young plants and the photosynthetic activity of mature plants, to the production of fruits and seeds. 

The identification and cloning of various genes for enzymes that enhance the biosynthesis of GB has been reported, and many lines of transgenic plants have been developed that express GB-biosynthetic genes from bacteria and plants. The various transgenic plants accumulate GB at a variety of levels and exhibit enhanced tolerance to various types of stress (Table **1**). Most lines of plants that have been engineered to synthesize GB are derived from plants that are natural non-accumulators of GB and the transgenic plants accumulate only low levels of GB. Within this group of plants, the greatest accumulation of GB was found in *codA*-transgenic rice plants (5.3 mmol g^-1^ FW; [68]. In a natural accumulator of GB, maize, the highest level of GB accumulated in *betA *transgenic plants was 5.7 mmol g^-1^ FW in leaves [69]. This level was higher than that in wild-type (WT) plants subjected to drought stress, indicating that transgenic plants were able to accumulate larger amounts of GB than the maximum amount in WT plants (Table **1**).

Waditee *et al.* [70] transformed *Arabidopsis *with genes for GSMT and SDMT from *Aphanothece halophytica *(designated *ApGSMT *and *ApSDMT*, respectively). They reported that the amounts of GB accumulated were higher than those in plants transformed with genes for choline oxidizing enzymes. However, the maximum levels of GB in their transgenic plants were close to 2.0 mmol g^-1^ FW, when plants were supplied with 0.1 m NaCl and 5 mm glycine. Thus, the combined actions of ApGSMT and ApDMT do not appear to be more useful than those of cholineoxidizing enzymes for engineering the biosynthesis of GB in plants. Su *et al.* [71] generated several lines of GB-producing transgenic rice plants, in which the *cox *gene for choline oxidase from *Arthrobacter pascens*, fused to a chloroplast targeting sequence, was expressed under the control of a stress-inducible promoter (SIP) or the promoter of a gene for ubiquitin (UBI), which was constitutively active. The highest level of accumulation of GB (2.6 mmol g^-1^ DW) in SIP lines that had been grown under saline conditions was not as high as that in UBI lines (3.1 mmol g^-1^ DW). Therefore, the use of SIP was no more effective for production of GB than the constitutively active UBI promoter. However, saline growth conditions enhanced the accumulation of GB by as much as 89% in the SIP lines, whereas a maximum increase of only 44% was seen in UBI lines. In spite of lower concentrations of GB, the stress tolerance of the SIP lines was significantly higher than that of the UBI lines, suggesting that the stress tolerance of the SIP plants was not attributable solely to increases in GB content. Early studies of natural accumulators of GB focused on the role of GB in maintaining the osmotic potential of cells, and the effective concentration of GB was assumed to be very high. However, the contribution of stress-induced GB and transgenetically accumulated GB to the total osmotic potential of cells is small and does not fully explain the associated increases in stress tolerance. In all of the previously mentioned lines of transgenic plants, levels of GB were below the range reported for plants that produce GB naturally (Table **1**).

With respect to possible advantages of the modification of chloroplast genomes, as distinct from nuclear genomes, in applications to agriculture, there are two reports of the genetic engineering of plastid genomes for GB synthesis. Genetic engineering of the chloroplast genome of carrot, which resulted in the expression of a gene for BADH from spinach, conferred strong tolerance to salt stress [72]. The highest level of GB was close to 100 mmol g^-1^ DW, which is the highest level reported in GB-accumulating transgenic plants to date. Zhang *et al.* [73] described the genetic transformation of the plastid genome of tobacco plant with the gene for CMO from sugar beet. Levels of GB in the leaves of the resultant tobacco plants ranged from 0.2 to 0.5 mmol g^-1^ FW, and GB appeared to be localized exclusively in chloroplasts. Although considerable efforts have been made to increase overall levels of GB in transgenic plants, reported levels of GB are still relatively low when compared to levels in natural accumulators of GB after their exposure to abiotic stress (Table **1**). Moreover, we do not yet understand why it has not been possible to achieve higher levels of GB in transgenic plants. There are two known factors that can limit the accumulation of GB in chloroplasts of transgenic plants: the availability of endogenous choline [74] and the transport of choline across the chloroplast envelope [75]. Huang *et al.* [74] introduced the metabolic steps for oxidation of choline to GB into three diverse species – *Arabidopsis thaliana*, *Brassica napus *and tobacco (*Nicotiana tabacum*). In these species, exogenous supply of choline significantly increased the level of GB accumulation, suggesting that choline supplement is required for the enhancement of GB levels in transgenic plants. Furthermore, the establishment of a model for the labelling kinetics of choline metabolites has revealed that the import of choline into chloroplasts limits GB synthesis in these compartments [75]. Furthermore, Nuccio *et al.* [76] found that the activity of PEAMT was 30 to 100 times lower in tobacco compared with spinach, suggesting that it may be the main reason why there is a limitation in the endogenous choline supply in tobacco plants, non-GB-accumulators. In plants, the biosynthesis of choline occurs exclusively in the cytosol [75]. Their research has provided the first example of engineered biosynthesis of choline, and has demonstrated that GB accumulation can be significantly increased in transgenic plants that normally accumulate GB only to very limited levels [77]. Therefore, it is likely that increased activity of PEAMT in non-GB-accumulators may further increase the level of GB accumulation in the transgenic plants. Further choline oxidase (*codA*) gene from *Arthrobacter globiformis *was transferred into *Brassica compestris* L. spp. Chinensis using Agrobacterium mediated transformation method [78]. The plants of transgenic line 1 (L1) showed significantly higher net photosynthetic rate (*P*_n_) and *P*_n_ recovery rate under high (45 °C, 4 h) and low temperature (1 °C, 48 h) treatments, and higher photosynthetic rate under high salinity conditions (100, 200, and 300 mmol/L NaCl, respectively) than the wild-type plants. Thus transgenic Brassica plants showing resistance to multiple abiotic stresses was a result of increased glycinebetaine accumulation [78]. Similarly transgenic tobacco plants expressing the *OsBADH1* gene were developed under the control of a promoter from the maize ubiquitin gene [79]. Three homozygous lines of T_2_ progenies with single transgene insert were chosen for gene expression analysis. RT-PCR and western blot analysis results indicated that the *OsBADH1* gene was effectively expressed in transgenic tobacco leading to the accumulation of glycine betaine. Transgenic lines demonstrated normal seed germination and morphology, and normal growth rates of seedlings under salt stress conditions. Therefore the results indicated that *Os BAGH1* is an excellent candidate gene for development of transgenic plants showing tolerance to increased osmotic stresses. In another attempt BADH gene was transformed into alfalfa (*Medicago* *sativa* L.) through Agrobacterium-mediated transformation method, and salt tolerance of the transgenic progenies was assessed [80]. The transgenic plants grew vigorous in salt stress condition, whereas the wild type plants was retarded and did not survive after cradle. The relative electrical conductivity and malondialdehyde (MDA) contents in the T_1_ transgenic plants were lower, but peroxidase (POD) and superoxide dismutase (SOD) activities were higher than those of the wild type plants. Thus the above results demonstrated that introduction of BADH gene into Alfalfa resulted in increased tolerance to salinity [80]. Recently, a chloroplastic BADH gene from *Spinacia oleracea* (*SoBADH*) was transferred into the sweet potato cultivar Sushu-2. The overexpression of *SoBADH* in the transgenic sweet potato improved tolerance to various abiotic stresses, including salt, oxidative stress, and low temperature [81]. The increased BADH activity and GB accumulation in the transgenic plant lines under normal and multiple environmental stresses resulted in increased protection against cell damage through the maintenance of cell membrane integrity, stronger photosynthetic activity, reduced reactive oxygen species (ROS) production, and induction or activation of ROS scavenging by the increased activity of free radical-scavenging enzymes. The above experiment reveals that the enhancement of GB biosynthesis in sweet potato is an effective and feasible method to improve its tolerance to abiotic stresses.

## CONCLUSION

In conclusion, GB accumulation could contribute to osmoregulation in natural accumulators; however, osmoprotection seems to be responsible for tolerance to abiotic stresses in transgenic plants. Extensive work on GB has suggested its varied roles in plants.

Possible mechanisms of GB-enhanced tolerance to various types of abiotic stress include:

1. stabilization by GB of the highly ordered structures of certain complex proteins to prevent denaturation when plants or plant cells are exposed to stress conditions;
2. induction by GB of the expression of specific genes that encode reactive oxygen species (ROS)-scavenging enzymes and subsequent depression of levels of ROS in plant cells; and
3. prevention by GB of the accumulation of excess ROS, resulting in protection of the photosynthetic machinery from the combined effects of light stress and other kinds of stress, as well as of ion-channel proteins and the integrity of cell membranes.

The increased production of glycine betaine (GB) improves plant tolerance to various abiotic stresses without strong phenotypic changes, providing a feasible approach to improve stable yield production under unfavorable conditions. New evidences suggest the contribution of differentially expressing endogenous genes in GB mediated stress tolerance in plants. Further work would establish whether the transcriptome changes are direct targets of GB or are product of metabolic adjustment in transgenic plants.

## Figures and Tables

**Table 1:** Genetic Engineering of Plants for Accumulation of GB.

Target Crop	Gene	Source	Remarks	Reference
Alfalfa *Medicago sativa L)*	BADH betaine aldehyde dehydrogenase	*E. coli*	The expression of foreign *BADH* gene enhanced the salt tolerance in transgenic alfalfa.	[80]
	BADH betaine aldehyde dehydrogenase	*E. coli*	Transgenic alfalfa plants grown under 9% NaCl grew well; while non-transgenic control plants turned yellowish in color, wilted, and eventually died.	[82]
*Arabidopsis(Arabidopsis thaliana)*	*CodA *choline oxidase	*Arthrobacter globiformis*	Plants tolerated 0 - 4 M NaCl for 48 h and 0.1 M NaCl for 20 d	[83, 84]
	*CodA *choline oxidase	*Arthrobacter globiformis*	Tolerant to cold and heat at 0 and 55°C, respectively	[85, 86]
	*CodA*/c*ox *choline oxidase	*Arthrobacter globiformis*	Increased salt, drought and freezing tolerance	[74]
	*CodA *choline oxidase	*Arthrobacter globiformis*	Enhanced tolerance to salt stress at the reproductive stage	[87]
	*ApGSMT + ApDMT*	*Aphanothece halophytica*	Tolerance to salinity and chilling	[70]
Carrot (*Dacus carota* )	BADH betaine aldehyde dehydrogenase	*E. coli*	Growth of transgenic plants at 400 mM NaCl	[72]
Cotton (*Gossypium* *hirsutum*	*bet A*	*E. coli*	Increased drought tolerance was observed in plants introduced with bet A gene	[88]
*Eucalyptus* *globulus*	*CodA *choline oxidase	*Arthrobacter globiformis*	Transgenic plants showed increased salinity tolerance	[89]
Japanese persimmon (*Diospyros kaki*)	*CodA *choline oxidase	*Arthrobacter globiformis*	Successful regeneration and salt tolerance at 0.1 M NaCl	[90]
*Mustard *(*Brassica juncea L.*)	*CodA *choline oxidase	*Arthrobacter globiformis*	Transgenic plants showed better growth and seed germination under salt stress	[91]
*Mustard (Brassica campestris * L. spp. *chinensis*	*CodA *choline oxidase	*Arthrobacter globiformis*	Transgenic plants showed higher net photosynthetic rate (*P*_n_) and *P*_n_ recovery rate under high (45 °C, 4 h) and low temperature (1 °C, 48 h) treatments, and higher photosynthetic rate under high salinity conditions (100, 200, and 300 mmol/L NaCl, respectively) than the wild-type plants.	[92]
*Potato *(*Solanum tuberosum*)	*CodA/cox *choline oxidase	*Arthrobacter globiformis*	Transgenic plants showed increased tolerance against salt drought and oxidative stress	[93]
*Potato *(*Solanum tuberosum*)	*SoBADH*	*Spinach *(*Spinacea oleracea*)	The BADH activity in the transgenic potato plants was between 10.8 and 11.7 U. The tolerance of transgenic BADH plants against abiotic stress was increased with increase in BADH activity	[94]
* Rice (Oryza sativa L)*	modified *bet A *choline dehydrogenase	*E. coli*	Salt and drought tolerant at 0.15 M NaCl and low relative humidity	[18]
	*CodA *choline oxidase	*Arthrobacter globiformis*	Salt tolerance at 0.15, 0.1 M NaCl and cold tolerance at 5 °C	[68]
	*CodA *choline oxidase	*Arthrobacter globiformis*	Salt stress tolerance	[95]
	*CodA/cox *choline oxidase	*Arthrobacter globiformis*	Stress tolerance in transgenic plants could not be explained whether it was due to increase in GB content.	[71]
	*CodA/cox *choline oxidase	*Arthrobacter globiformis*	Up regulation of several stress responsive genes	[96]
Sweet potato (*Ipomoea batatas*)	*SoBADH *betaine aldehyde dehydrogenase	*Spinach (Spinacea oleracea)*	Increased multiple abiotic stresses tolerance without causing phenotypic defects.	[81]
Tobacco* (N. tabacum)*	*bet A *choline dehydrogenase	*E. coli*	Better growth of transgenic plants at 0.3 M NaCl	[97]
	*bet A and bet B*	*E. coli*	Increased tolerance to salt stress	[98]
	*OsBADH1*	*Oryza sativa L*	Increased osmotic stress tolerance	[79]
	*CMO*	*Spinacea oleracea*	Increased salinity stress tolerance	[76]
	*SoBADH*	*Spinacea oleracea*	Transgenic plants showed increased tolerance to high temperature stress	[99]
	*SoBADH*	*Spinacea oleracea*	Transgenic plants showed increased tolerance to high salinity stress	[100]
	*BADH/Se NHX1*	*Saliconia europaea L.*	Simultaneous introduction of *BADH* and *Se NHX1 *gene resulted in increased accumulation of GB hence increased salinity tolerance.	[101]
*Tomato *(*Lycopersicon esculentum)*	*CodA *choline oxidase	*Arthrobacter globiformis*	Chloroplastic GB resulted in increased tolerance against chilling and oxidative stress.	[102]
	*CodA *choline oxidase	*Arthrobacter globiformis*	Transgenic tomato plants expressing cod A gene showed increased salt and water stress tolerance	[103]

## ABBREVIATIONS

GB: = Glycine Betaine
CMO: = Choline Monooxygenase
BADH: = NAD^+^-dependent Betaine Aldehyde Dehydrogenase
CDH: = Choline Dehydrogenase
COD: = Choline Oxidase
GSMT: = Glycinesarcosine Methyltransferase
SDMT: = Sarcosine Dimethylglycine Methyltransferase
